# Child, adolescent, and caregiver mental health difficulties and associated risk factors early in the COVID-19 pandemic in South Africa

**DOI:** 10.1186/s13034-022-00499-2

**Published:** 2022-08-11

**Authors:** Jenny Bloom, Anusha Lachman, Ezethu Gaxo, Jace Pillay, Soraya Seedat

**Affiliations:** 1 Department of Psychiatry, Faculty of Medicine and Health Sciences, Stellenbosch University, Private Bag X1 , Matieland, 7602 South Africa; 2 Department of Educational Psychology, Faculty of Education, University of Johannesburg, Johannesburg, South Africa

**Keywords:** COVID-19, Pandemic, Mental health, Strengths and Difficulties Questionnaire, Depression Anxiety Stress Scale, Child, Adolescent, Youth

## Abstract

At the onset of the COVID-19 pandemic in early 2020 in South Africa, many safety measures were implemented to protect the lives of the population. Ironically, these same safety measures have negatively impacted on the lives of children and their caregivers resulting in increased mental health problems. This study forms part of the multicountry Co-SPACE (COVID-19: Supporting Parents, Adolescents and Children during Epidemics) study that explores how families are coping during the COVID-19 pandemic, and what caregivers can do to help support their children’s mental health. This paper aims to gain a better understanding of the mental health status of families specifically in South Africa in the early onset of the pandemic during restrictive lockdown measures, and identify certain risk factors that might contribute towards deteriorating mental health. Two hundred and fifty-four South African parents and carers of children and adolescents completed an online survey about their child’s mental health as well as their own mental health during and post- hard lockdown in South Africa. Data collection took place over the period of the first and second waves of the COVID-19 pandemic in South Africa. Results showed that children experienced significantly higher mental health problems than adolescents (*p* = 0.016). Younger children were particularly negatively affected by lockdown and had more mental health problems than adolescents (*p* = 0.023); including emotional problems (*p* = 0.017), misconduct (*p* = 0.030), and hyperactivity (*p* = 0.001). Additionally, the presence of special educational needs/neurodevelopmental disorders (SEN/ND) was associated with more mental health problems (*p* = 0.001). Surprisingly, single parent households, which is another well-known risk factor showed no differences in mental health problems compared to nuclear families. There was also a reciprocal relationship between parental/carer mental health and child/adolescent mental health, with higher level of endorsement of mental health problems in children by parents/caregivers who themselves associated with higher levels of depression, anxiety and stress (all *p*’s < 0.001). These results highlight the dramatic impact that COVID-19 had on children, adolescents and parents in South Africa early in the pandemic, and emphasises the need for specific support structures to be implemented within the SEN/ND community, as well as for younger children and single parent households.

## Background

The COVID-19 pandemic has caused serious and unexpected disruptions to the lives of people around the world. These include sudden and unforeseen social isolation and lifestyle changes, the consequences of which are only recently beginning to present. While children and adolescents are considered at a lower risk of developing severe disease from COVID-19 infection compared to older individuals, children are not indifferent to the devastating consequence of the impact of the disease on the adults around them [1]. Additionally, measures that have been put in place to try and minimise spread of the virus have invariably impacted on the lives of children, adolescents and their families. As has been reported internationally, hard lockdown regulations, strict social distancing, the abrupt withdrawal from school, social life, and outdoor activities, increased rates of domestic and gender based violence, parental stressors, and loss of loved ones have all contributed to an increase in stress and anxiety levels [2–4]. In a low-and middle- income country (LMIC) such as South Africa, there are additional psychosocial and environmental stressors that impact on the mental health and well-being of children and their families. These include multiple risk factors such as high rates of parental mental illness, substance misuse, poverty, intimate partner violence and comorbid physical health conditions [5, 6].

While most COVID-19 related mental health research on children and adolescents has focused on high income settings, little is known about the impact of the pandemic on the mental health of children and adolescents in the South African context. Prolonged school closures and home confinement during a disease outbreak can have negative effects on children’s physical and mental health [7]. Children were exposed to longer screen time, less physical activity, poorer diets, and social isolation leading to irregular sleep patterns and mood responses [7]. Stress responses include fears of infection, frustration, boredom, lack of personal space and financial family stressors that cause more prolonged effects on mental wellbeing [5, 7]. Prolonged isolation at home exposes vulnerable children, especially those with existing mental health challenges or disabilities, to increased levels of abuse [4]. A child’s normal psychological development depends on routines, social interactions and friendships [4], thus being quarantined and isolated can break routines and add extra difficulties to an already challenging situation for all children and adolescents, particularly those with special needs or pre-existing psychiatric difficulties [8]. Indeed, children and adolescents who have experienced mental health problems before the pandemic may feel the effects of the pandemic more acutely and need additional support [9, 10].

Understanding the psychological impact of the pandemic on youth is crucial to target effective support where it is most needed, particularly where many children and adolescents are already facing known risk factors, such as in low income [11, 12] and single parent [13–15] households, and children with special educational needs (SEN) and neurodevelopmental disorders (ND) that require both health and educational support [16, 17]. Finally, gender and age are also known to influence the development of mental health problems. In primary school, boys are more likely to have any mental disorder (most commonly behavioural problems) than girls; however, in secondary school boys and girls are equally likely to have any mental disorder, but adolescent girls display higher rates of emotional disorders [18]. Finally, the impact of the pandemic may have differentially impacted different age groups, with adolescents being more self-sufficient at home schooling than children who would have relied heavier on their parents for this, placing an even bigger burden on already stressed parents during lockdown, including having to deal with remote learning, home office working, and endless house chores [19]. Research has shown that while younger children may be clingier or regress in behaviour during quarantine, older children may become more anxious, angry, restless and withdrawn [4]. For those living under conditions of precarity, without computers, mobile telephones, tablets, or a steady supply of electricity, access to the internet is often unavailable or prohibitively expensive. These individuals with limited or no internet access faced disadvantages, such as being uninformed as to the latest knowledge of the spread of the virus, or the latest guidelines issued by authorities; they may also be deprived of social support that is obtained through social media that allows the public to stay in touch with family, friends, and colleagues [20]. In comparison, those with easy access to the internet and data were likely to experience significantly fewer difficulties during lockdown, including access to news and online mental health, contact with others, and opportunities to work from home [20].

In 2021 it was found that only 76% of the South African population were internet users [21]. This lack of access to both the internet and smart devices may also have accentuated the educational disadvantage brought about by home-schooling being compromised, increasing the burden for potential mental health risk. In fact, research from the United States has found that young people, particularly secondary school pupils, experienced significant disruption and adversity during the pandemic and are experiencing a mental health crisis [38]. The authors describe these findings as a “cry for help”, underscoring the high degree of stress experienced by families during the pandemic [38]. Similarly, research conducted in LMIC’s including Ethiopia, Côte d’Ivoire and Lebanon has shown significant multidimensional, gender- and age-specific psycho-emotional effects due to the COVID-19 pandemic and government containment measures on adolescents [40]. Indeed, the pandemic combined with the public health response to the pandemic appear to be worsening pre-existing age vulnerabilities for adolescents, specifically in terms of anxiety and stress [40].

This paper reports on the state of mental health of children and adolescents, as well as their parents or carers, early in the pandemic during and after hard lockdown (i.e., alert level 5) in South Africa and assesses differences in mental health based on (i) child gender, (ii) household income (i.e., living in poverty or not)/family composition (i.e., single adult family or not), and (iii) the presence of SENs/NDs. The results reported in this paper are representative of the first and second waves of the COVID-19 pandemic in South Africa. This period included a hard lockdown which lasted the month of April 2020 and included the following restrictions: a national state of disaster and drastic measures to contain the spread of the virus and save lives, immediate travel restrictions and the closure of schools, social distancing and mask wearing, all social gatherings prohibited, tobacco and alcohol sales prohibited, and everyone to work from home if possible excluding emergency services.

## Methods

### Participants

Two hundred and fifty-four parents and caregivers based in South Africa, aged 18 years and older, completed the online survey over a period of 10 months from May 2020 to February 2021. Each one of the 254 responses represented a unique household. If participants had more than one school-going child, they were asked to choose one ‘index’ child to report on. The children whom they cared for ranged in age from 4 to 18 years. One hundred and ninety-three (76.0%) of the respondents resided in urban areas, while the remainder resided in rural areas.

### Recruitment

The study was advertised widely through various media (including television, radio and social media platforms such as Facebook and Twitter) and via professional organisations such as the South African Depression and Anxiety Support Group (SADAG), NECT (National Education Collaboration Trust), South African Society of Psychiatrists, South African Association of Child and Adolescent Psychiatrists and Allied Health Professionals, Occupational Therapy Association of South Africa, and The Psychological Association of South Africa and various NGOs and charities.

### Procedure

Parents/carers provided online informed consent after reading the online participant information sheet. If the participant was over 18 years of age and consented, they were directed to the survey link which allowed for electronic completion of the questionnaire using REDCap software.

### Ethical considerations

Ethical approval for this study was provided by the Stellenbosch University Health Research Ethics Committee (reference HREC1-2021–14,968). Data was collected anonymously and participants were recruited on a voluntary basis with no incentives or compensation for their participation. Informed consent was obtained for all participants.

## Measures

### Demographic characteristics

Parents/carers reported on their own and their child’s age, gender, and ethnicity as well as on their total household income, lifestyle, family composition, residential location and work status. Following Waite and colleagues [3], and due to the typical differences in patterns of child and adolescent mental health and differences in educational experience, we dichotomised age at baseline 4–10 year olds (children) and 11–18 year olds (adolescents). A household income below R30,000 per month was categorised as a ‘low household income’, while R30,000–R100,000 per month was categorised as a ‘middle household income’ and anything over R100,000 per month was a ‘high household income’. Finally, parents/carers were asked whether their child was diagnosed with a SEN/ND and if so, whether he/she had social, emotional and mental health difficulties, or communication and interacting difficulties, or cognition and learning difficulties, and finally, sensory and/or physical needs.

### The Strengths and Difficulties Questionnaire (SDQ)

The Strengths and Difficulties Questionnaire (SDQ) is a brief behavioural screening questionnaire for 4–17 year olds, and was used to measure the mental health of children and adolescents in the survey [22]. The SDQ covers the most important current domains of child psychopathology (i.e., emotional symptoms, conduct problems, hyperactivity-inattention, and peer problems) as well as personal strengths (i.e., prosocial behaviour) that can be completed by parents, teachers, and youth themselves. Several versions of the SDQ exist; however, for the purposes of this study we used the version that is answered on behalf of the children by their parents or carers. It is a 25-item scale describing positive and negative attributes of children and adolescents. It is comprised of 5 subscales, with 5 items in each subscale. Each item is scored on a 3-point scale with 0 = ‘not true’, 1 = ‘somewhat true’, and 2 = ‘certainly true’. Higher scores on the prosocial subscale reflect strengths, whereas higher scores on the other four subscales (i.e., the emotional symptoms subscale, the conduct problems subscale, the hyperactivity-inattention subscale, and the peer problems subscale) reflect difficulties. A total difficulties score can also be calculated by summing the scores on the emotional symptoms, the conduct problems, the hyperactivity-inattention, and the peer problems subscales (scores range from 0 to 40). The SDQ has been translated into 51 languages, and extensively validated in many Western and developing countries [23], but not in South Africa. In an African context the overall reported Cronbach alpha scores ranged from 0.18–0.89; however, Hoosen and colleagues [24] expressed concern about the robustness of the SDQ given the lack of reporting on validation in African studies. de Vries and colleagues [25] state that globally, the SDQ may be useful as an ‘in-country’ instrument, but they caution researchers when using it as a ‘cross-country’ comparative measurement tool. They advise that further validation work is required to compare the normative cut-off values with gold standard clinical diagnostic instruments. For a more detailed interpretation of SDQ scores on the parent completed SDQ see Table 1 below.

**Table 1:** Cut-off scores for the SDQ

Score	Normal	Borderline	Abnormal
Total difficulties score	0–13	14—16	17–40
Emotional problems score	0–3	4	5–10
Conduct problems score	0–2	3	4–10
Hyperactivity score	0–5	6	7–10
Peer problems score	0–2	3	4–10
Prosocial score	6–10	5	0–4

### The Depression Anxiety Stress Scale (DASS)

 The Depression Anxiety Stress Scale (DASS) is a set of three self-report scales designed to measure the negative emotional states of depression, anxiety and stress. This scale was used to assess the mental health of parents/carers in the survey. As outlined by Lovibond and Lovibond [26] the Depression scale assesses dysphoria, hopelessness, devaluation of life, self-deprecation, lack of interest/involvement, anhedonia, and inertia. The Anxiety scale assesses autonomic arousal, skeletal muscle effects, situational anxiety, and subjective experience of anxious affect. While the Stress scale is sensitive to levels of chronic non-specific arousal and assesses difficulty relaxing, nervous arousal, and being easily upset/agitated, irritable/over-reactive and impatient. The DASS21 is a shortened version of the DASS and contains 21 items, or 7 items per scale [27]. Subjects are asked to use a 4-point severity/frequency scale to rate the extent to which they have experienced each state over the past week. Each item is rated on a 4-point scale with 0 = ‘Did not apply to me at all’, 1 = ‘Applied to me to some degree, or some of the time’, 2 = ‘Applied to me a considerable degree, or a good part of time’, and 3 = ‘Applied to me very much, or most of the time’. Scores for Depression, Anxiety and Stress are calculated by summing the scores for the relevant items. When scoring the DASS21 one must multiply the scores by 2, to ensure that the scores are comparable to the original DASS. Higher scores suggest higher rates of depression, anxiety and stress. A study by Dreyer, Henn & Hill [28] investigated the psychometric properties of the DASS21 in a non-clinical South African sample. Results showed that the DASS is a valid and reliable instrument for research purposes in South African work settings. For a more in depth interpretation of DASS scores please see Table 2.

**Table 2:** Cut-off scores for the DASS tool (multiply DASS21 scores by 2)

Severity	Depression	Anxiety	Stress
Normal	0–9	0–7	0–14
Mild	10–13	8–9	15–18
Moderate	14–20	10–14	19–25
Severe	21–27	15–19	26–33
Extremely severe	28 +	20 +	34 +

### Data analysis

All analyses were carried out in SPSS (version 27). SDQ caseness categories were calculated using syntax downloaded from: https://www.sdqinfo.org/a0.html. To assess the mental health status of both children and parents, SDQ and DASS scores were examined in relation to well known risk factors such as: child age (child vs adolescent), household income (low, middle or high income), family composition (single parent household, or not) and SEN/ND (present or not).

For the risk factor *child age*, SDQ scores were analysed using a 2 (gender: male vs female) × 2 (age: child vs adolescent) between subjects factorial ANOVA across each of the five SDQ scales (emotional problems, conduct problems, hyperactivity, peer problems and prosocial). While DAS scores were analysed with independent samples t-tests comparing parent/carer DASS scores across the three DASS subscales (i.e., depression, anxiety and stress) in relation to the child’s age.

The risk factor *household income* analysed SDQ scores using a 2 (age: child vs adolescent) × 3 (income: low vs middle vs high) between subjects factorial ANOVA, while DAS scores were analysed using a one-way ANOVA.

*Family composition* represents whether a household is supported by a single adult or not. Due to the unequal sample numbers across the groups in the SDQ scores, family composition was analysed independently by removing child age. Thus, an independent-samples t-test examined whether family composition affected children’s mental health, as reported by their parents/carers. This was also done for each of the five SDQ subscales. Independent-samples t-tests were also conducted on the DASS scores and subscales.

The final risk factor was whether *SENs/NDs* (including social or emotional, communication, cognitive and sensory disorders) were present in the child or not. Due to the small number of children and adolescents who presented SENs or NDs, we were again unable to stratify by age. Thus, independent-samples t-tests were used to determine differences in total mental health scores in both the SDQ and DAS scores.

Finally, a Pearson’s correlation coefficient was computed to assess the relationship between reported child mental health (i.e., total SDQ score) and parent/carer mental health (i.e., depression, anxiety, and stress levels).

## Results

### Participant sample

Two hundred and fifty-four parents and caregivers completed the online survey. The vast majority, 246 (96.9%), had access to outside space. The majority of the children were in the care of their biological parents, 234 (92.1%), while 6 (2.4%) were fostered and 2 (0.8%) were adopted. For further information on the demographic of the sample please see Table 3.

**Table 3:** Sample* socio-demographic characteristics*

	Children (4-10yrs) *n* = 118 (46.5%)	Adolescents (11-18yrs) *n* = 127 (50.0%)	Undisclosed *n* = 9 (3.5%)	Full sample *n* = 254
Child				
Child mean age (SD)	7.81 (1.63)	13.8 (2.17)	–	10.91 (3.57)
Child gender				
Male	70 (59.3%)	67 (52.8%)	2 (0.8%)	139 (54.7%)
Female	47 (39.8%)	60 (47.2%)	5 (2.0%)	112 (44.1%)
Other/Unknown	1 (0.8%)	–	2 (0.8%)	3 (1.2%)
Parent/carer				
Parent gender				
Male	10 (18.5%)	14 (11.0%)	3 (1.2%)	27 (10.6%)
Female	108 (91.5%)	112 (88.2%)	5 (2.0%)	225 (88.6%)
Other/unknown	–	1 (0.8%)	1 (0.4%)	2 (0.8%)
Parent/carer education				
No qualifications	1 (0.8%)	3 (2.4%)	–	4 (1.6%)
Completed primary school (13yrs)	–	4 (3.1%)	–	4 (1.6%)
Completed secondary school (16yrs)	1 (0.8%)	9 (7.1%)	–	10 (3.9%)
Matriculated	17 (14.4%)	26 (20.5%)	4 (1.6%)	47 (18.5%)
Undergraduate degree/professional qualification	41 (34.7%)	32 (25.2%)	1 (0.4%)	74 (29.1%)
Postgraduate degree	57 (48.3%)	53 (41.7%)	2 (0.8%)	112 (44.1%)
Prefer not to say	1 (0.8%)	–	2 (0.8%)	3 (1.2%)
Employment status				
Working full-time	59 (50.0%)	58 (45.7%)	7 (2.8%)	124 (48.8%)
Working part-time	12 (10.2%)	11 (8.7%)	–	23 (9.1%)
Self-employed	26 (22.0%)	33 (26.0%)	–	59 (23.2%)
Full-time parents	9 (7.6%)	8 (6.3%)	–	17 (6.7%)
In school	2 (1.7%)	2 (1.6%)	–	4 (1.6%)
Unemployed	9 (7.6%)	15 (11.8%)	–	24 (9.4%)
Other	1 (0.8%)	11 (8.7%)	2 (0.8%)	3 (1.2%)
Family				
Family composition				
Single adult household	6 (5.1%)	16 (12.6%)	-	22 (8.7%)
Multiple adult household	109 (92.4%)	103 (81.1%)	6 (2.4%)	218 (85.8%)
Prefer not to say	3 (2.5%)	8 (6.3%)	3 (1.2%)	14 (5.5%)
Household income				
High (> R100 000 p.m.)	24 (20.3%)	15 (11.8%)	–	39 (15.4%)
Middle (R30 000 – R100 000 p.m.)	45 (38.1%)	52 (40.9%)	1 (0.4%)	98 (38.6%)
Low (< R30 000 p.m.)	30 (25.4%)	44 (34.6%)	6 (2.4%)	80 (31.5%)
Prefer not to say	19 (16.1%)	16 (12.6%)	2 (0.8%)	37 (14.6%)
Location				
Province				
Western Cape	51 (43.2%)	44 (34.6%)	5 (2.0%)	100 (39.4%)
Gauteng	36 (30.5%)	26 (20.5%)	1 (0.4%)	63 (24.8%)
Eastern Cape	10 (8.5%)	19 (15.0%)	1 (0.4%)	30 (11.8%)
Mpumalanga	6 (5.1%)	13 (10.2%)	–	19 (7.5%)
Kwa-Zulu Natal	9 (7.6%)	8 (6.3%)	–	17 (6.7%)
North West	2 (1.7%)	7 (5.5%)	1 (0.4%)	10 (3.9%)
Limpopo	3 (2.5%)	7 (5.5%)	–	10 (3.9%)
Free State	1 (0.8%)	2 (1.6%)	–	3 (1.2%)
Prefer not to say		1 (0.8)	1 (0.4%)	2 (0.8%)

*SEN/ND* special educational needs/neurodevelopmental disorders

This study also recorded mental health information for both the child and the parent/carer. These included whether the child had an SEN/ND and the type of SEN/ND as well as whether the child or the parent/carer had been diagnosed with any other mental health condition such as depression or anxiety, or diagnosed with a neurodevelopmental disorder such as autism spectrum disorder (ASD) or attention deficit hyperactivity disorder (ADHD). See Table 4 for more detail.

**Table 4:** Reported mental health status of children and their parents/carers

	Children (4-10yrs) *n* = 118 (46.5%)	Adolescents (11-18yrs) *n* = 127 (50.0%)	Undisclosed *n* = 9 (3.5%)	Full sample *n* = 254
Child SEN/ND	8 (6.8%)	24 (18.9%)	9 (3.5%)	32 (12.6%)
Social-emotional	1 (0.8%)	3 (2.4%)	1 (0.4%)	5 (2.0%)
Communication-interaction	2 (1.7%)	1 (0.8%)	1 (0.4%)	4 (1.6%)
Cognition-learning	4 (3.4%)	7 (5.5%)	–	11 (4.3%)
Sensory-physical needs	2 (1.7%)	2 (1.6%)	–	4 (1.6%)
Child mental health and ND				
Depression/anxiety/other	2 (1.7%)	12 (9.4%)	–	14 (5.5%)
ASD/ADHD	8 (6.8%)	24 (18.9%)	–	32 (12.6%)
Parent/carer mental health and ND				
Depression/anxiety/other	27 (22.9%)	21 (16.5%)	1 (0.4%)	49 (19.3%)
ASD/ADHD	3 (2.5%)	2 (1.6%)	2 (0.8%)	7 (2.8%)

*ASD* autistic spectrum disorder. *ADHD* attention deficit hyperactivity disorder

### The Strengths and Difficulties Questionnaire (SDQ)

#### Child gender and age

**Fig. 1:**
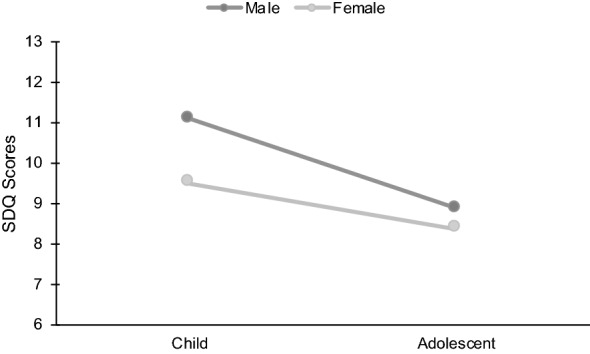
The effects of age and gender on mental health

There were significant differences in mental health scores between children and adolescents, such that children had higher mental health scores on the SDQ scale than adolescents, *F*(1, 240) = 4.88, *p* = 0.028, η^2^_p_ = 0.020 (see Fig. 1). There was no main effect of gender, *F*(1,240) = 1.89, *p* = 0.171, η^2^_p_ = 0.008, and no interaction between child age and gender on mental health scores, *F*(1,240) = 0.511, *p* = 0.476, η^2^_p_ = 0.002.

*Emotional problems scale* Children had higher scores for emotional problems than adolescents; however, this difference was not significant, *F*(1, 240) = 3.44, *p* = 0.065, η^2^_p_ = 0.014. There was also no significant gender difference in emotional problems, *F*(1, 240) = 0.605, *p* = 0.437, η^2^_p_ = 0.003, and no interaction between child age and child gender, *F*(1, 240) = 2.17, *p* = 0.142, η^2^_p_ = 0.009.

*Conduct problems scale* There was a significant difference in scores on the conduct problems scale between children and adolescents, with higher conduct problems endorsed in children than adolescents, *F*(1, 240) = 4.748, *p* = 0.030, η^2^_p_ = 0.019. There was no difference in conduct problem scores by gender, *F*(1, 240) = 2.071, *p* = 0.151, η^2^_p_ = 0.008, and there was no interaction between child age and child gender, *F*(1, 250) = 0.002, *p* = 0.968, η^2^_p_ = 0.000.

*Hyperactivity scale* There was a significant difference between children and adolescents. Children were reported to be more hyperactive than adolescents, *F*(1, 240) = 10.91, *p* = 0.001, η^2^_p_ = 0.043. There was also a significant gender difference, with males being more hyperactive overall compared to females, *F*(1, 240) = 4.37, *p* = 0.038, η^2^_p_ = 0.018. There was no interaction between age and gender, *F*(1, 240) = 0.74, *p* = 0.391, η^2^_p_ = 0.003.

*Peer problems scale* There was no significant difference in peer problems reported between children and adolescents, *F*(1, 240) = 2.68, *p* = 0.103, η^2^_p_ = 0.011, or between males and females, *F*(1, 240) = 1.58, *p* = 0.209, η^2^_p_ = 0.007. There was also no interaction between child age and child gender, *F*(1, 240) = 2.81, *p* = 0.095, η^2^_p_ = 0.012.

*Prosocial scale* There was no main effect for child age. Both children and adolescents reportedly displayed high prosocial behaviour, *F*(1, 240) = 1.45, *p* = 0.230, η^2^_p_ = 0.006. There was, however, a significant main effect for gender, with females displaying more prosocial behaviours than males, *F*(1, 240) = 56.15, *p* = 0.014, η^2^_p_ = 0.025. Finally, there was no interaction between age and gender, *F*(1, 240) = 0.88, *p* = 0.350, η^2^_p_ = 0.00. For further details on SDQ scores across child gender and age, See Appendix 1.

#### Household income

A 2 (age: child vs adolescent) × 3 (income: low vs middle vs high) between subjects factorial ANOVA revealed no main effects for any of the variables. This suggests that neither low, middle or high income made any difference to children’s or adolescent’s mental health, all *p’s* > 0.05. There was also no significant interaction observed between the variables. The same was observed for the SDQ subscales. No main effects were observed for the emotional, conduct, hyperactivity, peer problems or prosocial scales, and there were no significant interactions, all *p’s* > 0.05. This suggests that the children’s mental health as reported by their parents/carers was not affected by their age, nor total household income. Please see Appendix 2 for the ANOVA results.

#### Family composition

There was no difference in mental health scores between children raised in single adult households and those with more than one adult in the household (*p* = 0.183). As reported by parents/carers, children raised in single adult households exhibited fewer emotional problems than those raised by more than one adult (*p* = 0.021), and these children also displayed more prosocial behaviours than those raised by more than one adult (*p* = 0.010). There was no difference in conduct problems, hyperactivity levels, or peer problems between those children raised in single parent households compared to those raised by more than one adult (all *p’s* > 0.05, see Table 5 for more detail).

**Table 5:** SDQ scores by family composition

	*M*		*SD*		*n*	*t*	*p*	Cohen’s *d*
	Single	Not single	Single	Not single				
Overall	8.04	9.78	5.91	5.95	242	− 1.34	0.183	0.29
Emotional problems	1.65	2.50	1.50	2.34	242	− 2.41	0.021	0.43
Conduct problems	1.35	1.57	1.64	1.66	242	− 0.61	0.544	0.13
Hyperactivity	3.26	3.63	2.47	2.74	242	− 0.62	0.533	0.14
Peer problems	1.78	2.09	2.30	1.76	242	− 0.76	0.446	0.15
Prosocial	9.00	8.02	1.31	1.76	242	2.59	0.010	0.63

#### Presence of SENs/NDs

Due to the small number of children and adolescents with SENs or NDs, we were not able to stratify by age. Participants with SENs/NDs had significantly higher mental health scores than those without (*p* < 0.001).

The effect of SENs/NDs (present or not) on mental health scores were examined more closely across the five SDQ subscales. As reported by parents and carers, youth with SENs/NDs had higher emotional mental health scores (*p* = 0.003), were reported to be more hyperactive (*p* < 0.001), and experienced more peer problems (*p* = 0.038) than those without SENs/NDs. Finally, there was no difference in misconduct scores (*p* = 0.052), or prosocial behaviour between participants with and without SENs/NDs (*p* = 0.068, see Table 6 for more detail).

**Table 6:** SDQ scores of youth with and without SEN/ND

	*M*		*SD*		*N*	*t*	*p*	Cohen’s *d*
	SEN/ND	None	SEN/ND	None				
Overall	14.38	9.12	5.40	5.82	252	− 4.40	< 0.001	0.91
Emotional problems	3.69	2.30	2.71	2.23	252	− 2.95	0.003	0.61
Conduct problems	2.15	1.49	1.99	1.61	252	− 1.95	0.052	0.40
Hyperactivity	5.77	3.35	2.58	2.56	252	− 4.57	< 0.001	0.95
Peer problems	2.77	1.99	2.14	1.78	252	− 2.08	0.038	0.43
Prosocial	7.50	8.16	2.30	1.67	252	1.83	0.068	0.38

### The Depression Anxiety Stress Scales (DASS)

#### Child age

There were no differences in depression scores (*p* = 0.145), or anxiety levels (*p* = 0.133) between parents/carers who reported on children compared to those reporting on adolescents. However, parents/carers who reported on a child experienced significantly higher levels of stress compared to those who reported on an adolescent (*p* = 0.009). See Table 7 for more details.

**Table 7:** Parent/carer DASS scores by child age, family composition and SEN/ND present or not present in the child

	*M*		*SD*		*n*	*t*	*p*	Cohen’s *d*
Child age	Child	Adolescent	Child	Adolescent				
Depression	7.81	6.30	8.64	7.54	244	1.46	0.145	0.19
Anxiety	5.08	3.82	7.02	6.06	244	1.51	0.133	0.19
Stress	12.45	9.31	9.65	9.00	244	2.64	0.009	0.34
Family composition	Single	Multiple	Single	Multiple				
Depression	8.73	6.81	10.77	7.78	233	1.06	0.290	0.24
Anxiety	6.18	4.18	8.84	6.09	233	1.40	0.163	0.31
Stress	11.36	10.83	12.05	9.22	233	0.25	0.804	0.06
SEN/ND	Present	Not present	Present	Not present				
Depression	8.80	6.86	8.54	8.01	246	− 1.13	0.259	0.24
Anxiety	5.68	4.32	8.85	6.28	246	− 0.98	0.327	0.21
Stress	13.92	10.55	9.86	9.36	246	− 1.70	0.091	0.36

#### Household income

There were no differences in levels of depression (*F*(2,211) = 0.624, *p* = 0.537, η^2^_p_ = 0.005), anxiety (*F*(2,211) = 1.154, *p* = 0.318, η^2^_p_ = 0.01), or stress levels (*F*(2,211) = 1.34, *p* = 0.264, η^2^_p_ = 0.013 across low, middle or high income groups. See Table 8 for descriptive statistics.

**Table 8:** One-way ANOVA comparing parent/carer DASS scores by household income

	*M*			*SD*		
	Low	Middle	High	Low	Middle	High
Depression	6.51	7.12	8.26	8.19	7.29	9.02
Anxiety	5.31	3.94	3.90	7.90	4.97	6.09
Stress	9.69	11.43	12.46	9.34	9.38	9.31

#### Family composition

There were no statistically significant differences in levels of depression (*p* = 0.290), anxiety (*p* = 0.163) or stress (*p* = 0.804) between single parents and those who had assistance raising their families. See Table 7 for more details.

#### Presence of SENs/NDs

The following section examines how having a child with an SEN/ND affects parent/carer mental health. No differences were found in depression (*p* = 0.259), anxiety (*p* = 0.327) or stress (*p* = 0.091) levels between parents/carers who had children with SENs/NDs and those who did not. See Table 7 for more details.

### Correlations between SDQ and DASS scores

Moderate, positive correlations were found between total child/adolescent SDQ scores and parent/carer DASS scores for depression (*r*(244) = 0.37, *p* < 0.001), anxiety (*r*(244) = 0.35, *p* < 0.001), and stress (*r*(244) = 0.45, *p* < 0.001). Parents/carers who experienced higher levels of depression, anxiety and stress also reported higher mental health problems overall in their children/adolescents.

## Discussion

This study set out to assess how families coped during the COVID-19 pandemic, by focusing on mental health symptoms in children (based on parent/carer report) and their parents/carers in the first year of the COVID-19 pandemic in South Africa. The main findings were as follows: children were reported to have more mental health problems than adolescents overall, they were also reported to show higher misconduct and hyperactivity than adolescents. Children and adolescents from single led households were reported to have fewer emotional problems and higher prosocial behaviour. Children and adolescents with SENs/NDs were reported to have more mental health problems than those without SENs/NDs, this included more emotional, hyperactivity and peer problems. Moreover, parents/carers who reported on younger children also displayed higher levels of stress. Finally, parents/carers who experienced higher levels of depression, anxiety and stress also reported higher levels of mental health problems overall in their children/adolescents.

Examining the SDQ results brought a few important findings to light, particularly the risk in the mental health of children (aged 4–10 years old). Parents and carers endorsed high levels of mental health problems in children and adolescents, especially children. This finding is worrying as previous research has pointed to the fact that adolescents usually experience more mental health issues than children [29, 30]. It highlights the impact that the COVID-19 pandemic and consequent restrictions might have had on children who are perhaps too young to understand the meaning of the pandemic, compared to adolescents. Younger children are a lot more reliant on their parents throughout the day including support with education and monitoring, entertaining and providing for them; all of which would increase family stress. Contrary to this, adolescents may have been more independent during lockdown with better access to their peers on social media, online chats, and gaming [3]. This justification might also explain why parents/carers of younger children had higher DASS stress scores than those with adolescents. It seems that while lockdown had a negative impact on young children’s mental health, having to care for and raise young children during lockdown also took a toll on the stress levels of parents and carers. These heightened stress levels could be explained by challenging behaviours, with the relationship between stress and difficult behaviours being reciprocal [31]. The impact of lockdown on family stress and peer relationships is certainly a point of interest for future research, which should assess whether the challenges associated with the early lockdown period persist over time, or whether they resolve when children and adolescents are able to return to some of their normal activities.

Family composition played a role in the mental health of children and adolescents. A single parent household is a common risk factor for poor mental health, which is partly due to material disadvantage [13, 14]. It was found that those raised in single parent households exhibited fewer emotional problems and displayed more prosocial behaviour than those raised by more than one adult. These findings are very interesting, and contradictory to research that has shown adolescents from one parent families report more emotional problems including lower self-esteem, more symptoms of anxiety and loneliness, more depressed mood, and more suicidal thoughts than children from intact families [15]. While most of the research concerning single parenthood has focused on the disadvantages faced by children raised in the absence of a father, there is now a need for greater emphasis on assessing the strengths and resiliency of single parent homes. A possible explanation for these contrary findings might be that the relationship fostered between a single parent and child results in fewer emotional problems, and more prosocial behaviour. Research has shown that high levels of balanced connectedness in the parent–child relationship predicts high levels of empathy and prosocial behaviour [32]. It might be that single parents are more likely to share a balanced connectedness with their children. They might also monitor or structure their children’s behaviours more closely instead of relying on a spouse or co-parent to do so. As single parents may not have another adult to share parental duties with, they may be more aware of their child’s whereabouts, activities, and friendships, and it has been shown that this type of parental knowledge promotes prosocial behaviour among youth [33]. One further explanation could be that in single parent families, exposure to spousal/partner and gender based violence may be minimal; thus, children may have more positive mental health experiences. Future studies might look closer at the type of relationship fostered between single parents and their children.

Parents and carers with a child with a SEN/ND reported more mental health problems in the child, including emotional, hyperactivity/inattention, and peer problems. These findings are supported by other work which has found that low mood and distress are experienced more severely within the SEN/ND community [34], and families who raise SENs/NDs face more stressors, which can influence family relationships negatively making patient and empathetic parenting challenging at times [35]. This reciprocal relationship can be seen in the positive correlation between the SDQ and DASS scores. There was a significant relationship between higher depression, anxiety, and stress levels amongst parents/carers and endorsement of more mental health problems in their children. Further studies should investigate whether this relationship is in fact reciprocal, or merely a projection from a parent who perhaps is not fully aware of their child/adolescent’s feelings. It is also important to note the possibility that someone who is experiencing high levels of depression, anxiety and/or stress would conceive that their child felt the same, and there may be some contagion of negative mood state in the home. Another consideration is sampling bias, which confounds comparison of parents/carers of children with SENs/NDs versus children without SENs/NDs. Parents/carers were asked to choose only one of their children to report on and so may have had a child with an SEN/ND but instead chose to report on a different child. Future studies should take this into consideration and perhaps include a question that asks parents if they have a SEN/ND child? If yes, then to report on that child, or if no, to report on any child.

Surprisingly, household income, a well-known risk factor, was not associated with a child or adolescent’s mental health scores. On reflection, the household income boundaries used in this study might have been too broad. As of May 2021, the average salary in South Africa was approximately R23 500 per month. Thus, the condition of “R0–R30 000 per month” incorporated a below average, average and above average salary into the same group. Future studies examining South African household income should use salary ranges that are more representative of South African earnings. Another surprising finding was the lack of mental health issues reported by the parents/carers themselves during the beginning of the pandemic. Those who reported on a child (4–10 years old) also reported feeling higher stress levels; however, household income, family composition and the presence of a SEN/ND made no difference to the parents’ depression, anxiety or stress levels as reported by themselves on the DASS. A potential explanation for this is that parents may have underreported poorer mental health to be viewed in a more positive light. It is also possible that parents/carers may not have been aware of the full extent of any mental health symptoms in their children/adolescents, particularly emotional symptoms [3].

In addition to the limitations stated above, other limitations of this study include the use of self-report measures, which are susceptible to biases and limitations. Subjects may not be entirely truthful in their responses, instead answering in a more socially acceptable manner. Parents/carers were asked to choose an ‘index’ child to report on, which might have resulted in them choosing the child whose mental health was most adversely affected by the pandemic. Conversely, they might have chosen the most resilient child to appear more socially acceptable. Future studies should consider including a question to further identify why the parent/carer chose that specific child to report on. Self-report measures also assume that people can introspect, while some people may not be able to assess themselves accurately. The wording of the questions may also have different meanings or interpretations to different people. Response bias can affect the quality of the answers given. For example, question order and whether they relate to recent or significant experiences can affect the responses given by participants [36]. Future studies might consider combining self-report data with either behavioural or physiological data to gain a more multi-modal assessment.

Finally, the original Co-SPACE study spearheaded by Waite and Creswell in 2020 used the SDQ to assess child and adolescent mental health. This study attempted to replicate the parent study as closely as possible, in terms of design, data collection and analysis, to identify similarities and differences with how families coped with the pandemic globally. However, the Child Behaviour Checklist (CBCL) is a widely used questionnaire to assess behavioural and emotional problems and would also have been a very useful tool to use in a sub-Saharan African context.

## Conclusion

There is already a mental health care gap among children and adolescents [37], and the COVID-19 pandemic will widen this gap if we are not vigilant in providing the right type of support where it is needed the most. Recent research has shown that the COVID-19 pandemic and related restrictions severely impacted the mental health of adolescent girls and young women in South Africa, and despite the high burden of mental health challenges, policies and services in South Africa are sadly inadequate [39]. Results from this study show that both children and parents within the SEN/ND community, as well as single parent families, and parents/carers who are caring for young children under the age of 11 during lockdown would all benefit from additional support and assistance during the COVID-19 pandemic. These are potentially vulnerable groups who need particular attention. With the reopening of schools around the world, mental health resources could be leveraged in schools as they offer an important entry-point to integrate gender- and age-responsive psychosocial support into existing curricula [40]. The education sector had to adapt and respond to COVID-19 by delivering lessons virtually. This expansion of virtual platforms could also allow for the integration of psychosocial modules promoting mental well-being and referral of those children and adolescents in need of mental health support [40]. The advantages of digital tools include large scale service at low cost and largely in private, which reduces stigma. However, further efforts are needed to allow this aid to be accessed by all parts of society, including the more vulnerable, without exacerbating digital inequalities [40]. While the findings are not directly generalizable to other geographical contexts, the implications of these results may help people understand how families have been progressing through the pandemic, realising which measures work and which do not, and discovering what is particularly difficult for families and how to best help them during this time.

## Data Availability

The datasets used and/or analysed during the current study are available from the corresponding author on reasonable request.

## Appendix 1

The effect of child age and gender on a) emotional problems, b) conduct problems, c) hyperactivity, d) peer problems, and e) prosocial behaviour.

### Appendix 2

3 way ANOVA results

**Table:**

	Predictor	Sum of Squares	*df*	Mean Square	*F*	*p*	η_p_^2^
SDQ Total	(Intercept)	16,309.58	1	16,309.58	461.13	< 0.001	0.684
	hhincome	9.43	2	4.72	0.13	0.875	0.001
	childage	92.90	1	92.90	2.63	0.107	0.012
	hhincome x childage	15.00	2	7.50	0.21	0.809	0.002
	Error	7533.49	213	35.37			
Emotional	(Intercept)	994.41	1	994.41	182.95	< 0.001	0.462
	hhincome	6.67	2	3.34	0.61	0.542	0.006
	childage	17.14	1	17.14	3.15	0.077	0.015
	hhincome x childage	1.39	2	0.70	0.13	0.880	0.001
	Error	1157.75	213	5.44			
Conduct	(Intercept)	398.38	1	398.38	144.23	< 0.001	0.404
	hhincome	6.45	2	3.23	1.17	0.313	0.011
	childage	4.25	1	4.25	1.54	0.216	0.007
	hhincome x childage	1.71	2	0.85	0.31	0.735	0.003
	Error	588.33	213	2.76			
Hyperactivity	(Intercept)	2322.60	1	2322.60	336.11	< 0.001	0.612
	hhincome	10.73	2	5.37	0.78	0.461	0.007
	childage	24.50	1	24.50	3.55	0.061	0.016
	hhincome x childage	16.94	2	8.47	1.23	0.296	0.011
	Error	1471.90	213	6.91			
Peer problems	(Intercept)	785.24	1	785.24	227.96	< 0.001	0.517
	hhincome	6.07	2	3.03	0.88	0.416	0.008
	childage	2.29	1	2.29	0.66	0.416	0.003
	hhincome x childage	1.39	2	0.70	0.20	0.817	0.002
	Error	733.70	213	3.45			
Prosocial	(Intercept)	11,617.77	1	11,617.77	3803.13	< 0.001	0.947
	hhincome	8.53	2	4.27	1.40	0.250	0.013
	childage	0.97	1	0.97	0.32	0.574	0.001
	hhincome x childage	7.68	2	3.84	1.26	0.286	0.012
	Error	650.67	213	3.06			

## Abbreviations

ADHD: Attention deficit hyperactivity disorder
ASD: Autism spectrum disorder
Co-SPACE: COVID-19-Supporting Parents, Adolescents and Children during Epidemics.
COVID-19: Coronavirus disease 2019
DASS: Depression, Anxiety and Stress Scale
LMIC: Low-and middle- income country
SDQ: Strengths and Difficulties Questionnaire
SEN/ND: Special educational needs/neurodevelopmental disorders
